# Subject-Specific Tendon-Aponeurosis Definition in Hill-Type Model Predicts Higher Muscle Forces in Dynamic Tasks

**DOI:** 10.1371/journal.pone.0044406

**Published:** 2012-08-29

**Authors:** Pauline Gerus, Guillaume Rao, Eric Berton

**Affiliations:** 1 Centre for Musculoskeletal Research, Griffith Health Institute, Griffith University, Gold Coast, Australia; 2 Institute of Movement Sciences E-J Marey, Aix-Marseille Université, Marseille, France; University of Rochester, United States of America

## Abstract

Neuromusculoskeletal models are a common method to estimate muscle forces. Developing accurate neuromusculoskeletal models is a challenging task due to the complexity of the system and large inter-subject variability. The estimation of muscles force is based on the mechanical properties of tendon-aponeurosis complex. Most neuromusculoskeletal models use a generic definition of the tendon-aponeurosis complex based on *in vitro* test, perhaps limiting their validity. Ultrasonography allows subject-specific estimates of the tendon-aponeurosis complex’s mechanical properties. The aim of this study was to investigate the influence of subject-specific mechanical properties of the tendon-aponeurosis complex on a neuromusculoskeletal model of the ankle joint. Seven subjects performed isometric contractions from which the tendon-aponeurosis force-strain relationship was estimated. Hopping and running tasks were performed and muscle forces were estimated using subject-specific tendon-aponeurosis and generic tendon properties. Two ultrasound probes positioned over the muscle-tendon junction and the mid-belly were combined with motion capture to estimate the *in vivo* tendon and aponeurosis strain of the *medial head of gastrocnemius muscle*. The tendon-aponeurosis force-strain relationship was scaled for the other ankle muscles based on tendon and aponeurosis length of each muscle measured by ultrasonography. The EMG-driven model was calibrated twice - using the generic tendon definition and a subject-specific tendon-aponeurosis force-strain definition. The use of subject-specific tendon-aponeurosis definition leads to a higher muscle force estimate for the soleus muscle and the plantar-flexor group, and to a better model prediction of the ankle joint moment compared to the model estimate which used a generic definition. Furthermore, the subject-specific tendon-aponeurosis definition leads to a decoupling behaviour between the muscle fibre and muscle-tendon unit in agreement with previous experiments using ultrasonography. These results indicate the use of subject-specific tendon-aponeurosis definitions in a neuromusculoskeletal model produce better agreement with measured external loads and more physiological model behaviour.

## Introduction

The force produced by the muscle fibres is transmitted to the bones via the aponeurosis and tendon. The aponeurosis and the tendon are not rigid and therefore their lengths change in some proportion to applied loading. This elasticity of the aponeurosis and tendon is important in transmitting force from muscles to bone. For example, during an isometric contraction when the overall muscle-tendon unit length remains constant muscle force production results from the shortening of muscle fascicles while the tendon-aponeurosis complex stretches [1]. During a dynamic task such as walking, Ishikawa and al. [2] have used ultrasonography to quantify the in vivo behaviour of the muscle fibre with respect to the muscle-tendon unit length change. The muscle-tendon unit undergoes a stretching followed by a shortening, while the muscle fibre behaviour may be different. The muscle fibre could shorten or remain constant during the stretching of the muscle-tendon unit. The independent behaviour of the muscle fibre and muscle-tendon unit, called decoupling, is a result of the compliance of the aponeurosis-tendon unit. Decoupling reduces the muscle fibre velocity and thus directly affects the production of muscle fibre force. Furthermore, the decoupling within the muscle-tendon unit could be influenced by the intensity of tasks, with a decrease in decoupling related to an increase in task intensity [3], [4]. The tendon and the aponeurosis thus are important to the decoupling between the muscle fibre and the muscle-tendon unit, and to muscle force production during either static or dynamic tasks.

Neuromusculoskeletal (NMS) models are developed to estimate muscle forces important to the investigation of motor control and neuromusculoskeletal disorders. Developing accurate models is a challenging task because of system complexity and the presence of high inter-subject variability in many of the system components. The interaction between the muscle fibre and the tendon-aponeurosis complex is usually represented in a NMS model with a Hill-type muscle model. In a Hill-type muscle model, an elastic element (Series Elastic Element: SEE) representing the tendon-aponeurosis complex is in series with two parallel elements representing the muscle fibre. The series elastic element is usually defined by a generic force-strain relationship based on *in vitro* tests on tendon. [5]. The use of this generic definition is questionable for several reasons. First, the mechanical properties were measured in cadaveric specimens and do not reflect human *in vivo* behaviour [6]. Second, the generic definition is applied to models regardless of the nature of the task and the subject. Recent studies have shown a large variability in tendon mechanical properties depending on the sex [7], the type exercise [8], the age [9], and the loading rate (rate of force application to the tissue) [10], [11]. Finally, the generic definition is used to represent both the tendon and aponeurosis mechanical properties assuming their respective mechanical properties are equivalent. Given the recent advances in tendon characterization and the inter-subject variability, it is necessary to properly define the SEE in a NMS model in order to accurately estimate muscle forces.

The mechanical properties of the tendon and aponeurosis have previously been estimated for different muscles using ultrasonography during isometric contractions. Previous studies have found a similar strain of the tendon and aponeurosis [12], [13], and both a higher strain for the aponeurosis [14], [15] and tendon [16], [17]. The lack of consistent results may be explained by experiments being conducted on various muscles, with different methods, or by the large variability in muscle-tendon mechanics between individuals. Due to the variability in tendon and aponeurosis mechanical properties, it is necessary to represent the tendon and aponeurosis as two separate elements within a Hill-type muscle model. Lieber et al [18] developed a Hill-type muscle model which modelled the tendon and aponeurosis as two elements in series with the muscle fibre. The force-strain relationship of the tendon and aponeurosis was estimated from experiments on frog muscle. Importantly, the simulation of fixed-end contraction has shown that the mechanical definition of the SEE influenced the model outputs [18]. The attempt by Lieber et al. [18] to separate the tendon and aponeurosis was promising, but limited to animal experimentation and during fixed-end contraction.

The aim of this study was to investigate the influence of the tendon and aponeurosis considered as two separate elements in Hill-type muscle model defined by subject-specific mechanical properties on EMG-driven model outputs such as the muscle force estimations and muscle fibre behaviours during highly dynamic tasks. By using a simulation, Bobbert [19] has found that a Series Elastic Element more compliant increases the maximal squat jump height. Thus our hypothesis was that the use of a subject-specific tendon-aponeurosis force-strain relationship in a Hill-type muscle model will lead to higher estimates of muscle forces compared to the model estimates using a generic tendon force-strain relationship.

## Materials and Methods

### Experimental Design

Seven healthy males (age 25.9±1.6 years, body mass 72.7±9.8 kg, height 1.77±0.05 m) participated in this study. All participants gave informed written consent. The study was approved by the Ethical Committee of Aix-Marseille University and was conducted in accordance with the Declaration of Helsinki. Subjects performed two types of tasks to investigate the influence of subject-specific definition of the SEE on muscle forces and estimated fibre behaviours: (i) Isometric contractions to estimate the *in vivo* tendon-aponeurosis force-strain relationships, and (ii) running and hopping tasks.

For the isometric contractions, subjects were seated on the bench of a custom ergometer with the knee fully extended and the ankle joint at 0° dorsiflexion. This ankle angle was chosen to minimize effects from the passive joint moment and obtain large forces from the medial head of the gastrocnemius (GM) muscle. The right foot was set in a rigid shoe fixed to a plate with both joint and static torquemeter (CS3, Meiri, France) axis aligned. Subjects performed three 3-second maximal voluntary isometric plantar-flexion contractions (MVIC) with verbal encouragement and on-screen joint moment feedback. Then subjects performed ramp-up contractions (from rest to 100% MVIC) in minimal time with on-screen feedback. (Two trials were recorded for each condition with a resting time of 2 min. The contraction duration was chosen to be close to loading rates encompassed during running and hopping tasks.

**Figure 1:**
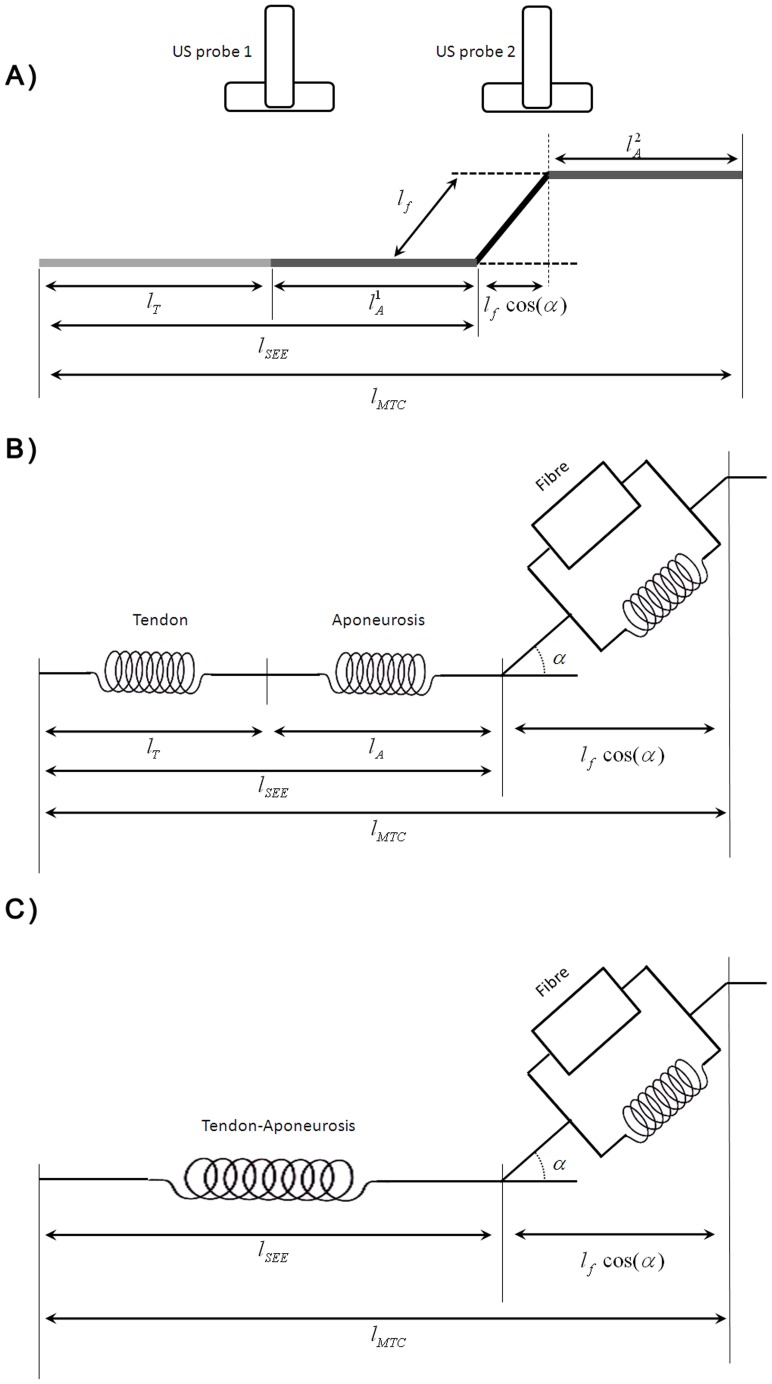
Illustration of method used to estimate Subject-Specific Tendon-Aponeurosis. (A) Geometric model of the muscle-tendon-aponeurosis complex that includes fascicle, tendon, and aponeurosis adapted from Fukashiro et al. [23]. The aponeurosis length is: 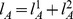
_._ Hill-type muscle model with the tendon and the aponeurosis in series (B) and a resulting Tendon-Aponeurosis element representing the tendon and aponeurosis in series (C).

During isometric contractions, the ankle joint net moment was recorded by the static torquemeter at 2000 Hz. Kinematic data were recorded synchronously at 125 Hz by a six-camera VICON system (Vicon Motion System, Lake Forest, CA). Three retroreflective markers were attached to an ultrasound probe located over the muscle-tendon junction (MTJ) in order to create a probe reference frame parallel to the image plane and further used to estimate the tendon length [11]. Additional markers were attached to the Achilles’ tendon insertion over the calcaneus, the first metatarsal head of the foot, the lateral and medial malleoli of the ankle, and the fibula head. The first 50 mm linear array ultrasound probe (BK-Med Pro-Focus) was positioned over the MTJ of the GM muscle, while a second 40 mm linear array ultrasound probe (BK-Med Ultra-view) was fixed over the mid-belly of GM muscle (Fig. 1) at the level of 30% of the lower leg length (i.e. from the popliteal crease to the centre of the lateral malleolus) and aligned approximately in the same plane as the muscle line action [20]. The probes were carefully attached to the leg using foam fixation and secured with elastic bandage. Images were recorded synchronously at 30 Hz with two image acquisition boards (National Instrument, PCI 1410). The synchronization of the ultrasound video acquisition with Vicon motion capture system was triggered by an analog signal (0–5V). In rest position, a single ultrasound probe was used to measure the tendon length, the fascicle length and the pennation angle for the *lateral head of the gastrocnemius* (GL), *medial head of the gastrocnemius* (GM), and *soleus* (SOL).

For the dynamic contractions, subjects performed hopping and running tasks. They performed two series of 15 continuous hops on the right leg over a force plate (Kistler, USA) at an approximate frequency of 2 Hz [21]. They were asked to hop in-place without shoes and with their hands on their hips. Then, subjects performed two running trials at a self-selected speed with their right foot landing on the force plate. Markers were attached over the insertion of the Achilles’ tendon on the calcaneus, the fifth and first metatarsal heads of the foot, the lateral and medial malleoli of the ankle, the fibula head, the lateral and medial condyles of the femur, the great trochanter for both legs, and the right and left anterior superior iliac spines. The coordinates of each marker was recorded at 125 Hz. EMG signals were recorded at 2000 Hz (Trigno Wireless, Delsys, USA) using surface electrodes placed over the muscles according to recommendations of the SENIAM group. Muscle activity for the GL, GM, SOL and tibialis anterior (TA) were recorded.

### Estimation of the Tendon and Aponeurosis Force-strain Relationships for the GM Muscle

Ultrasound images from the first probe were used to track the MTJ defined as the intersection between the GM muscle fascicles and the GM tendon. Raw images were processed by adjusting the contrast and then binarized using a graythreshold value. The MTJ displacements were tracked using the method presented by Korstanje et al. [22] with manual adjustment when necessary. All possible pixel displacements within the search region were evaluated with normalized cross-correlation, with the higher value used to determine the MTJ position on the next frame. For each frame, the tendon length was computed as the distance between the markers placed on calcaneus and the MTJ position in the global coordinate system using the probe reference frame [11].

The tendon strain of the GM muscle (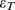) was defined as:
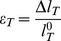
(1)Where 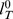
is the initial tendon length computed in the rest position and 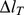
is the elongation of tendon.

**Figure 2:**
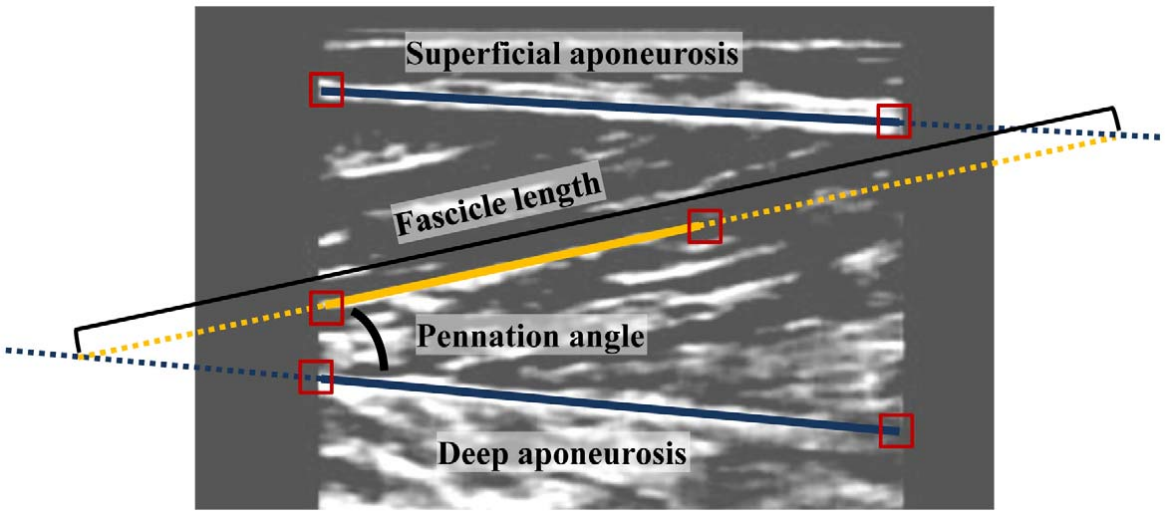
Illustration of method used to estimate the fascicle length and pennation angle. The proximal and distal ends of the superficial aponeurosis, deep aponeurosis, and muscle fascicle were tracked automatically at each frame. The missing part was estimated by linear interpolation.

The aponeurosis length was computed based on the geometric model (Fig. 1) presented by Fukashiro et al. [23] with muscle fascicle length, pennation angle, tendon length and muscle-tendon length as input at each time step. First, the muscle fascicle length (Lf) and the pennation angle (α) of GM muscle were computed using ultrasound data obtained from the second probe. The muscle fascicle length was defined as the distance between the intersection of the fascicle with the superficial and deeper aponeurosis. The proximal and distal ends of both superficial and deep aponeurosis were tracked automatically for each frame [22] (Fig. 2). Each aponeurosis was defined by linear interpolation between the proximal and distal end. This process directly accounts for changes in position of the aponeurosis during contraction and indirectly took into account the effect of the internal pressure of the muscle. Proximal and distal ends of each fascicle were tracked automatically for each following frame using the method presented by Korstanje et al. [22]. The entire muscle fascicle could not be seen on the US image, thus the missing part of fascicle was estimated by linear extrapolation of both aponeurosis and fascicle [24] (Fig. 2). The pennation angle was defined as the angle between the deeper aponeurosis and the muscle fascicle. The curvature of the fascicle was not taken into account. The possible error of this assumption is less than 6% [25]. The fascicle length and pennation angle were estimated for two fascicles and averaged at each time instance with the US probe placed over the mid-belly of GM muscle to account for variability amongst the fascicles. The averaged data were used to represent the entire muscle fibre. Shin et al. [26] have shown by using MRI data that there is no difference in muscle fascicle length along the GM muscle but that the pennation angle could change from the distal to the proximal region of GM muscle. The value of the pennation angle for the middle part of the GM muscle is the average of the distal and proximal regions. Sampling the mid-part of the GM muscle appears a reasonable way to represent the entire muscle.

To estimate the aponeurosis length, we hypothesized that the muscle fibre lengths and pennation angles estimated using ultrasonography in the muscle mid-belly were representative of the entire muscle behaviour [27].

The muscle-tendon length was computed from Opensim by using kinematic data of the isometric task. First, a geometric musculoskeletal model was scaled for each subject by using a static upright trial. Then, the markers positions obtained at each time during contraction were used in Opensim to estimate joint angles with the Inverse Kinematic Tool. Finally, the muscle-tendon length was computed at each time for all subjects and all trials [28].

The aponeurosis length was computed at each time using:
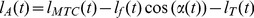
(2)Where 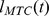
is the muscle-tendon length computed by Opensim at each time instance.

The aponeurosis strain of the GM muscle was defined as:
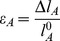
(3)Where 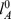
is the initial aponeurosis length estimated at the same instance as 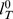.

Over the entire force production range the relative moment contribution of the GM to the ankle joint net moment and the moment arm of GM were assumed constant [29]. Consequently, the normalized force was computed as the measured joint moment normalized by its maximum value during the task. Finally, a second-order polynomial model was used to fit the tendon and aponeurosis force-strain relationship for the GM muscle.

### Estimation of the Tendon-aponeurosis Force-strain Relationship for Each Muscle

The tendon and the aponeurosis were considered as two elements in series (Fig. 1). These two elements in series could be represented by one element, henceforth called Tendon-Aponeurosis (T-A). In rest position, one ultrasound probe was used to measure the tendon length (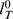), the fascicle length and the pennation angle for the GL, GM and SOL. The aponeurosis length in the rest position (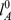) was estimated by using the geometric model described previously (Fig. 1). A muscle-specific ratio for the GL, GM and SOL was defined to represent the tendon length according to the T-A length such as:
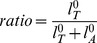
(4)


From this ratio, the strain for the tendon-aponeurosis complex is:
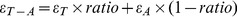
(5)

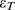
 represents the tendon strain of the GM muscle and 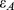 the aponeurosis strain of the GM muscle.

**Figure 3:**
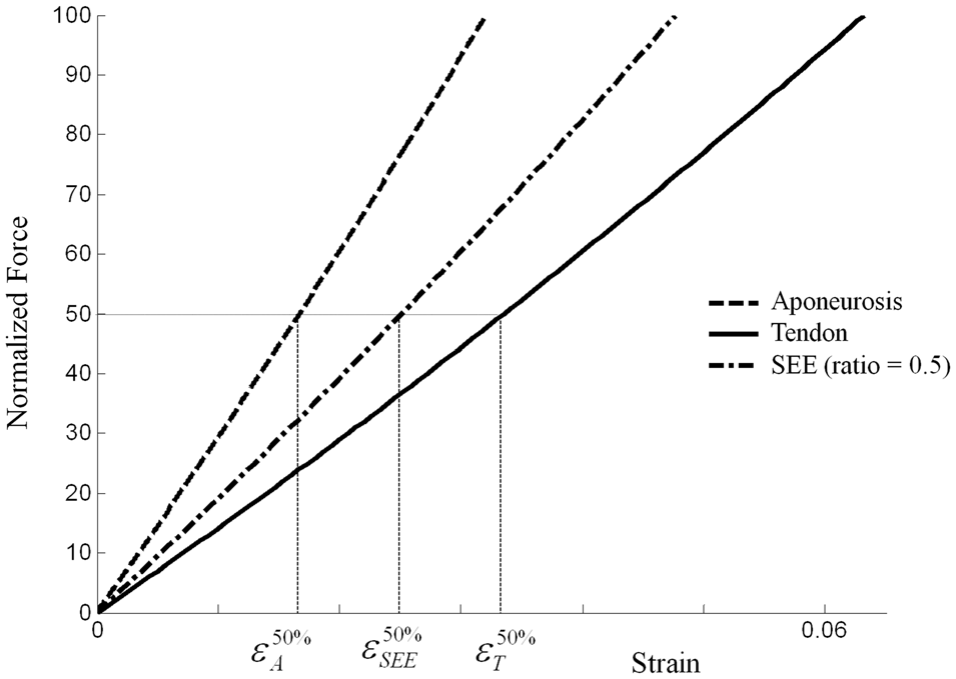
Estimation of Tendon-Aponeurosis force-strain relationship from tendon and aponeurosis individual force-strain relationship. At the same force level, the tendon and aponeurosis strain were estimated and combined with an experimentally-derived ratio to compute the Tendon-Aponeurosis strain (see eq. 5 in text).

At each force level, the T-A strain was estimated by using equation 5, the muscle-specific ratio and the corresponding tendon and aponeurosis strain for each subject. Fig. 3 illustrates the method used to estimate the T-A force-strain relationship based on the aponeurosis and tendon force-strain relationships and the muscle specific ratio.

### Calibration and Prediction Process of the EMG-driven Model

**Figure 4:**
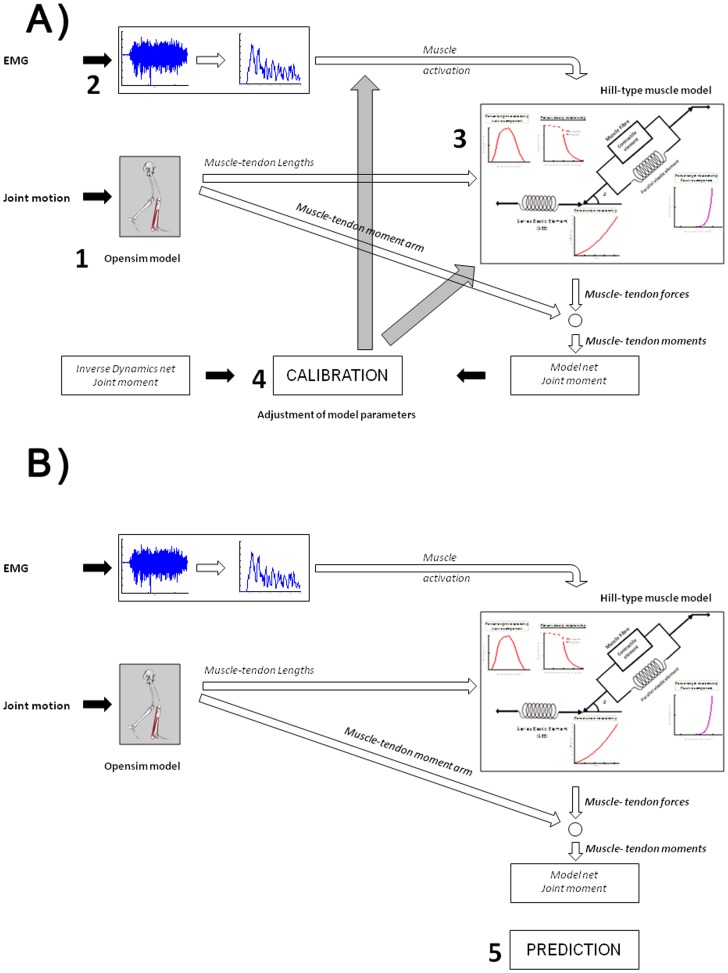
Description of EMG-driven model (A) Calibration and (B) Prediction process of the EMG-driven model. (1) represents the Anatomical Model used to estimate muscle-tendon lengths and moment arms, (2) an EMG-to-Activation Model to represent muscle activation dynamics, (3) a Hill-type Model to characterize muscle-tendon contraction dynamics and estimate the forces in the muscle-tendon complex, (4) a calibration process to tune model parameters based on ankle net joint moment computed by Inverse Dynamic, and (5) a prediction process to predict joint moment by using adjusted model parameters.

Once the T-A force strain relationship was estimated for each muscle and each subject, the next step was the muscle force estimation by using the EMG-driven model. The model used in the present study has already been extensively described [30], [31] and only a brief description is given here. The EMG-driven model used EMG, kinematic, anatomical, and net joint moment data to estimate muscle forces. The model consisted of five parts: (1) an anatomical model to estimate muscle-tendon lengths and moment arms, (2) an EMG-to-activation model to represent muscle activation dynamics, (3) a Hill-type model to characterize muscle-tendon contraction dynamics and estimate the forces in the muscle-tendon unit, (4) a calibration process to tune model parameters based on ankle net joint moment computed through inverse dynamics, and (5) a prediction process to compute joint moments by using adjusted model parameters (Fig. 4).

OpenSim (Simtk, Stanford, USA) was used to create an anatomical model and to scale this model based on the subject’s static marker measurements (more information in Delp et al. [29]). This model included four muscle-tendon units (GL, GM, SOL and TA). Based on kinematic measurements, the muscle-tendon lengths and flexion-extension moment arms at the ankle joint were estimated at each time instance for each subject and trial.

The raw EMGs were band-pass filtered (10–500 Hz), full-wave rectified, filtered using a zero-lag Butterworth low-pass filter (4th order, 6Hz cut-off frequency) and normalized by the maximal EMG value for each muscle obtained during isometric and dynamic tasks. Normalized EMG was transformed to muscle activation by combining a linear second-order differential equation to represent the electromechanical delay and a specific function to represent the non-linear relationship between EMG and muscle activation [29], [32].

The muscle activations and muscle-tendon lengths for each subject were used as inputs into a Hill-type muscle model to estimate muscle forces. In the Hill-type model, the muscle fibre was represented by a contractile element, characterized by a force-length-velocity relationship, in parallel with an elastic element, characterized by a passive force-length relationship. The SEE in series with the muscle fibre was characterised by a force-strain relationship (either generic or subject-specific). During the process of muscle force estimation, the muscle fibre length and velocity were estimated at each time instance such that the muscle fibre force is equal to the SEE force. Finally, muscle forces were multiplied by the muscle-tendon moment arms and summed to determine joint net moment.

Due to the large inter-subject variability, several model parameters need to be adjusted to accurately estimate the ankle joint net moment. During the calibration process, model parameters were adjusted using constrained numerical optimization which minimized the difference between the ankle joint net moment computed by the model (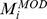) and the ankle joint net moment estimated through Inverse Dynamics (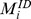) across the entire trial using the following objective function:
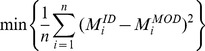
(6)


Two SEE definitions of the Hill-type model were used during the calibration process:

(1) The in-vivo subject-specific tendon-aponeurosis force-strain relationship obtained previously by using ultrasonography (henceforth referred to as SS T-A condition), and (2) the generic definition (henceforth referred to as Generic condition) given by:



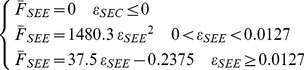
(7)with 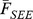 and 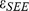 being the normalized SEE force and the SEE strain respectively [5].

Once the calibration process was completed on one trial of running and hopping, the adjusted model parameters were used to predict the ankle joint net moment using EMG and kinematics data from novel running and hopping trials. The predicted model moments were compared for SS T-A and Generic conditions. The data obtained from model predictions were further named *prediction*.

### Data Analysis

Only the prediction process data were analysed to evaluate the robustness of the EMG-driven model (opposite to the calibration process that is more a curve fitting process). The net joint moment predicted by the model was compared with the net joint moment computed by Inverse Dynamics. R^2^ represent the coefficient of determination used to indicate the closeness of fit between estimated and measured ankle net joint moment. Correlation between model and inverse dynamics moments has been used to investigate the ability of the NMS model to predict the net joint moment [30], [31]. The R^2^ is computed for each trial on the complete time history such as:
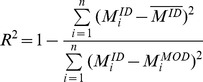
(8)

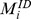
 represents the ankle joint net moment computed through inverse dynamics and used as reference, 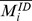
is the average of ankle joint net moment computed by inverse dynamics, and 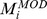 represents the ankle joint net moment computed by the EMG-driven model.

The residual mean square error (RMS_error_) reflects the magnitude of the error across the complete time history and is computed as:
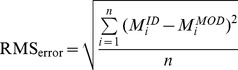
(9)


For each prediction trial, the following model outputs were analysed: maximal net joint moment estimated by the NMS model, maximal muscle forces, variation (maximum value–minimum value) in muscle fibre length, fibre velocity and SEE length.

Student’s paired *t* tests were conducted on the predicted model outputs described previously for GL, GM, SOL and TA muscles. A significance level of 0.05 was set *a priori* for all comparisons and Newman-Keuls Post-Hoc testing was used when necessary.

## Results

### Tendon-Aponeurosis Properties

**Figure 5:**
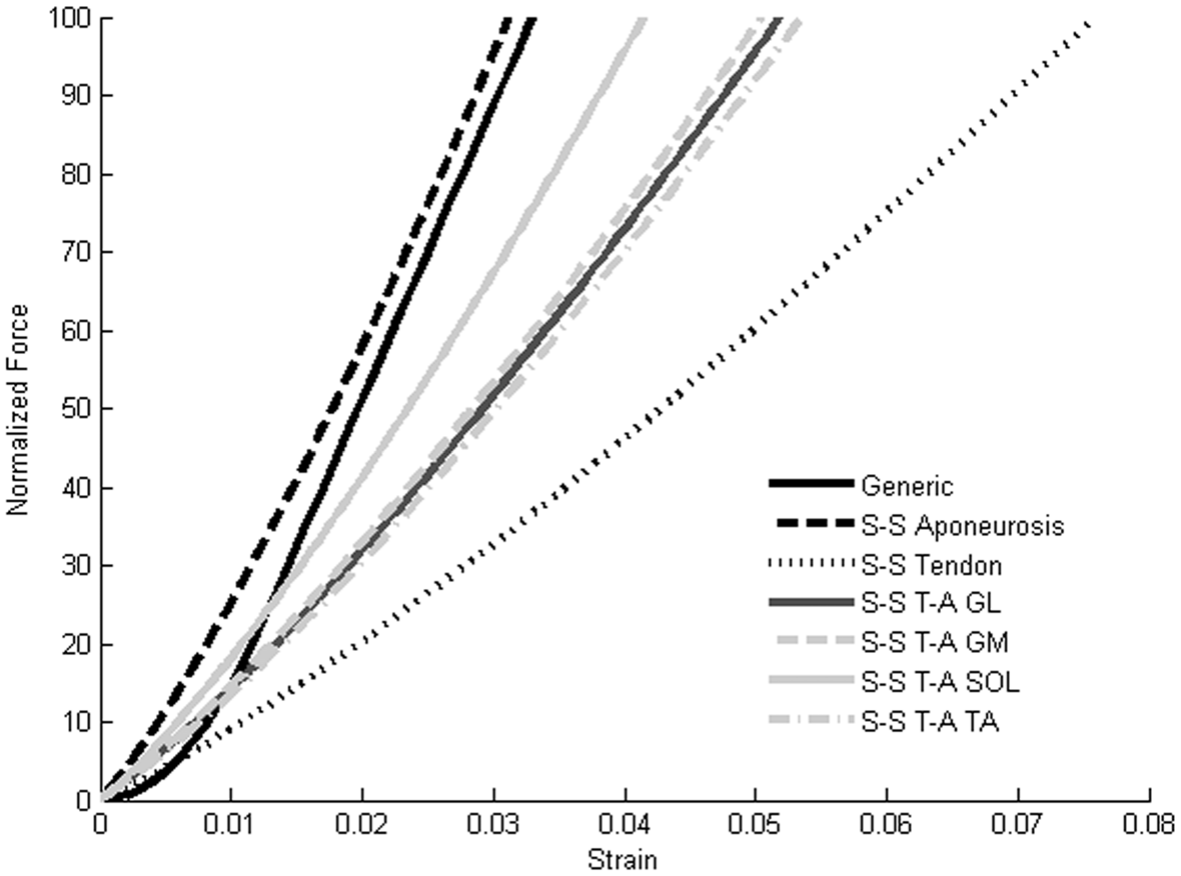
Example of input and output data of an EMG-driven model. Example of input data (Normalized EMG, muscle-tendon lengths and moment-arms) and output data (muscle forces and net ankle joint moment) for one subject during the contact phase of a hopping trial. The output data were obtained for both SEE definitions: the SS T-A and the generic.

The mechanical characterization of tendon and aponeurosis was performed on the GM muscle. The aponeurosis strain was lower than the tendon strain at the same force level, showing the aponeurosis was stiffer than the tendon. Additional measurements were necessary to estimate the ratio between the tendon length and tendon-aponeurosis length. This ratio was lower for the *soleus* muscle (0.23) whereas for the GM and GL muscles the ratio was close to 0.5 highlighting a tendon length equal to the aponeurosis length (Table 1). Whereas the aponeurosis was stiffer than the tendon, the small ratio of *soleus* leads to higher stiffness of the T-A unit compared to the three others muscles (Fig. 5). Finally, the SS T-A force-strain relationships showed higher compliance compared to the generic definition currently used in NMS model (Fig. 5).

**Table 1:** Summary of muscle-tendon geometric parameters estimated by US in rest position or from Maganaris et al. [6] and used to estimate the muscle specific tendon-aponeurosis force-strain relationships.

	GL	GM		SOL	TA	
**Initial Fascicle length (mm)**	70.2±2.9		59.5±3.6	43.8±4.9		–
**Initial pennation angle (°)**	13.2±3.2		19.4±1.7	19.4±1.2		–
**Initial tendon length (mm)**	222.1±32.9		209.9±34.0	61.4±14.6		–
**Initial aponeurosis length (mm)**	194.0±11.3		219.3±9.1	217.8±19.3		–
**Ratio**	0.53±0.05		0.49±0.05	0.22±0.04		0.5†

† represent the value used from Maganaris et al. [6].

### EMG-driven Model Inputs and Outputs

**Figure 6:**
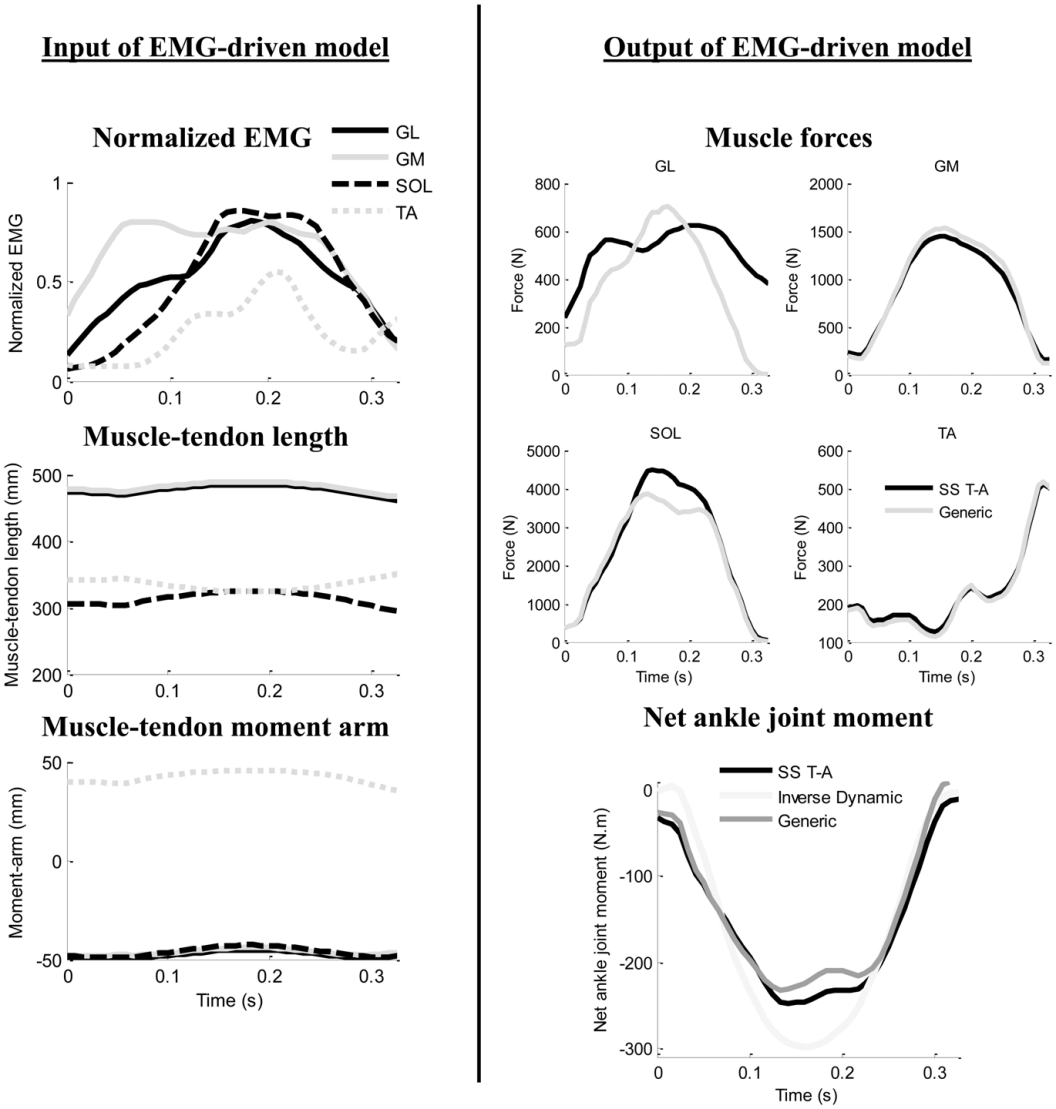
Force-strain relationships estimated by ultrasonography and used in Hill-type muscle model. Group averaged subject-specific tendon and aponeurosis force-strain relationships of the GM and subject-specific Tendon-Aponeurosis force-strain relationships for each muscle. The generic tendon force-strain relationship described by Zajac [5] and used for the generic calibration and prediction processes is also presented for comparison.

Figure 6 illustrates the data used as input to the EMG-driven model and the model outputs in terms of muscle forces and net ankle joint moment. The R^2^ for prediction process was significantly higher for SS T-A condition (0.70±0.14) than the Generic condition (0.66±0.16). The averaged RMS_error_ was 44.8±13.4 N.m and 44.8±14.6 N.m respectively for SS T-A condition and the Generic condition. The prediction of the minimal value of the net ankle joint moment was significantly lower for SS T-A condition (-166.2±53.4 N.m) than for the Generic condition (-145.7±42.2 N.m).

**Figure 7:**
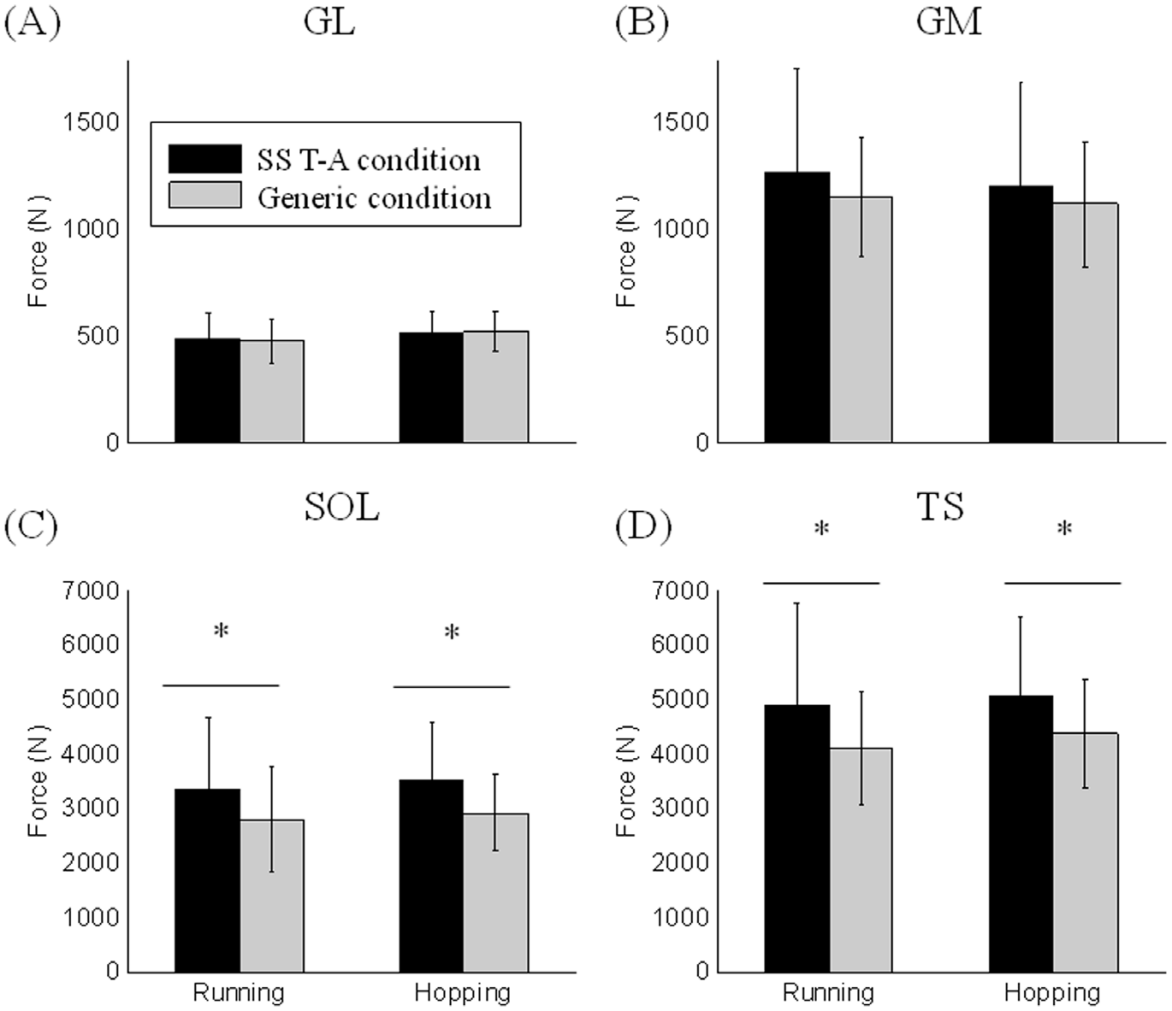
Comparison of maximal muscle force: SS T-A vs. Generic. Mean maximal force estimated by the EMG-driven model according to the condition, SS T-A and Generic for the GL (A), GM (B), SOL (C) and the *Triceps Surae* group (i.e., sum of GL, GM, and Sol muscles) (D). * indicates a significant difference between the conditions (SS T-A vs. Generic).

The use of subject-specific tendon-aponeurosis definition led to higher muscle force estimations for the plantar-flexor group than the generic condition (4988.9±1602.5 N vs. 4246.0±992.3 N) and the *soleus* muscle (3444.8±1136.7 N vs. 2870.5±803.6 N) (Fig. 7). No significant difference was found for the other three muscles.

The EMG-driven model computed the muscle fibre length and velocity to estimate the muscle force. Compared to the generic condition, the use of SS T-A definition leads to an increase of the SEE length variation and a decrease of the fibre length and velocity variations for the *soleus* muscle (Table 2).

**Table 2:** Summary of the behaviour of the muscle fibre and the tendon-aponeurosis unit in terms of variation of length and velocity.

	GL		GM		SOL	
	SS T-A	Generic	SS T-A	Generic	SS T-A	Generic
**Variation of fibre velocity (mm.s^−1^)**	563±13	655±184	476±139*	599±147*	524±136*	652±137*
**Variation of fibre length (mm)**	24±3	27±3	19±3	22±1	22±4*	25±3*
**Variation of SEE length (mm)**	6±2*	4±1*	10±4*	8±2*	10±3*	7±2*

* Significative difference between SS T-A and Generic.

**Figure 8:**
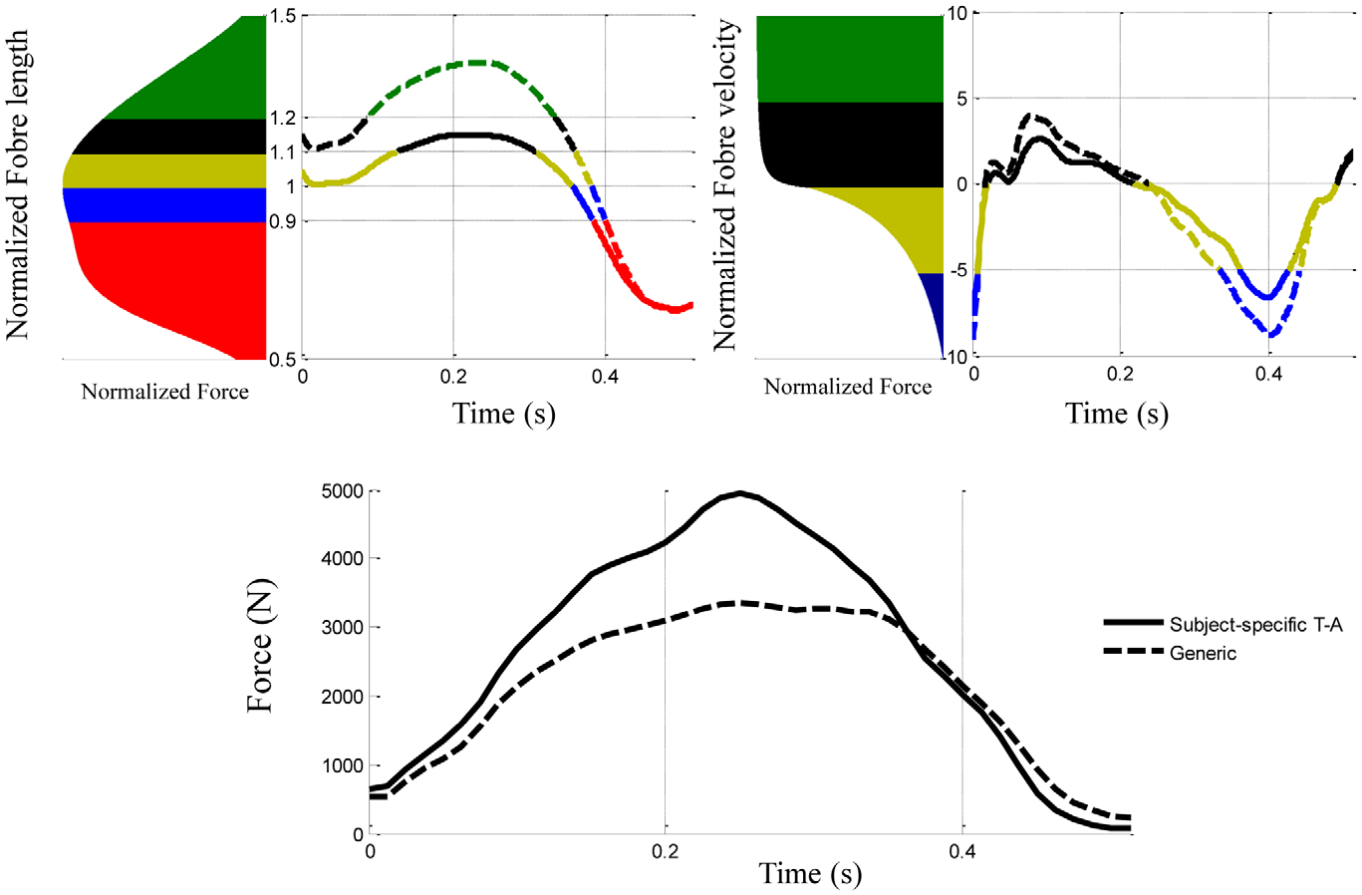
Normalized fibre length and velocity with corresponding muscle forces for the *soleus*. Trajectory of normalized fibre length and velocity for one subject corresponding to the *soleus*. The colours of the curve represent the different part of the force-length and force-velocity relationships cover by the normalized fibre length and velocity according to the SEE definition. The figure at the bottom represents the muscle forces according to the SEE definition.

**Figure 9:**
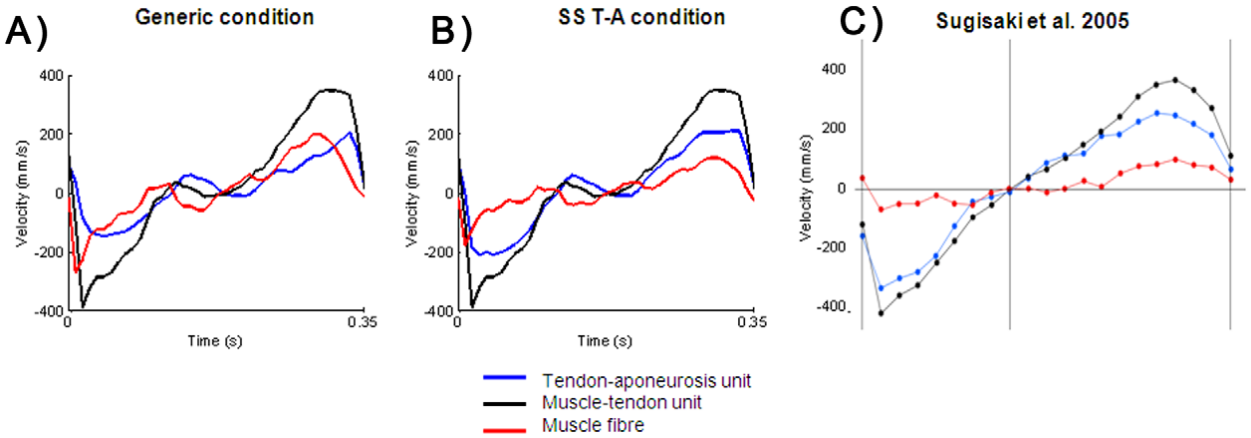
Decoupling behaviour between muscle fibre and muscle-tendon unit. Comparison of the velocity of the muscle fibre, the tendon-aponeurosis unit and the muscle-tendon unit from one subject on running tasks for the (A) generic and (B) SS T-A conditions and from (C) a previous experimentation with the muscle fibre velocity measured using ultrasonography. Note that the SS T-A condition leads to physiologically sound behaviour of the muscle fibre while the generic condition simply shared the muscle-tendon stretch over both the fibre and the SEE complex. Reprinted with permission.

## Discussion

In the present study, the influence of subject-specific tendon-aponeurosis properties in NMS models was investigated for variables such as the muscle forces and muscle fibre behaviour. The subject-specific tendon-aponeurosis was first estimated for the medial head of the gastrocnemius combining motion capture and two ultrasound probes, and then scaled for each muscle by using a ratio between the tendon length and the aponeurosis length in rest position. The subject-specific definition of tendon-aponeurosis unit was more compliant than the generic definition from Zajac [5] currently used in most NMS models. This subject-specific definition was then used as input into EMG-driven model to estimate muscle forces during hopping and running. The use of subject-specific tendon-aponeurosis in an EMG-Driven model leads to higher muscle force estimations.

### Mechanical Characterization of T-A

The mechanical characterization of the tendon and the aponeurosis emphasize that these two components have different mechanical properties, with the aponeurosis being stiffer than tendon. This difference in properties means both the tendon and the aponeurosis need to be defined separately. Given the different tendon and the aponeurosis stiffness, additional measurements of the GM, GL and SOL were performed to estimate the ratio between the tendon and aponeurosis lengths at rest. Based on this ratio, the tendon-aponeurosis force-strain relationship was scaled for each muscle and each subject. We hypothesized that the mechanical properties of the tendon and the aponeurosis are the same for all the studied muscles. This assumption seems reasonable as the tendon of the GM muscle has a common distal part with the GL and SOL muscles, suggesting a close behaviour. However, further studies on mechanical characterization of tendon and aponeurosis for all three plantar-flexor muscles will be necessary to validate this hypothesis. A constant ratio was used for the Tibialis Anterior muscle based on previous measurements [14]. The contribution of the TA to the ankle joint is small compared to the three other muscles and the lack of effect of SS T-A on TA muscle variables suggest that it’s most important to be focused on the three other muscles than the TA. The ratio was close to 0.5 for the GM and GL whereas the mean value was 0.23 for the SOL. This lower ratio for the soleus leads to tendon-aponeurosis characterization stiffer than the three others muscles but still less stiff than the generic condition currently used into EMG-driven model [7]. Taken together, these results suggest a clear difference in terms of mechanical properties of the SEE for the two conditions tested in the present study (i.e., generic vs. SS T-A).

### Muscle Force Estimations

Our results showed the importance of the SEE definition in muscle force estimations as the muscles forces and ankle net joint moment estimations were strongly affected during hopping and running tasks. The use of SS T-A definition in Hill-type muscle model leads to higher muscle forces for the *soleus* and the plantar-flexor group (i.e., sum of the soleus and the gastrocnemii muscles). The higher force for the plantar-flexor group explains the higher absolute value of ankle net joint moment computed by the EMG-driven model with the SS T-A definition. In addition, the use of SS T-A improves the prediction ability of the model by a 4% increase in the R^2^ value.

The effect of SEE definition in Hill-type muscle model has been previously investigated through simulations [19], [30], [33]. These simulations were focused on the determination of an arbitrary value of SEE compliance maximizing either the performance or the muscle force. They have shown that the use of less stiff SEE definition in Hill-type muscle model increases either the estimation of muscle force or the squat jump performance. Whereas simulations represent an average subject with an arbitrary choice of the SEE compliance, our study combined subject-specific patterns of muscle activations from EMG, kinematic motion and external ground reaction forces and subject-specific SEE definition. The results of our study are in agreement with these previous simulations showing that an increase of SEE compliance leads to estimate higher muscle forces during highly dynamic tasks. The generic Zajac definition appears to be too stiff to well represent the SEE into Hill-type muscle model. To our knowledge, the present study is the first to report specific T-A properties that are able to maximize the muscle force production during human movement.

### Decoupling Behaviour between Muscle Fibre and Muscle-tendon Unit

The force produced by the muscle fibre depends on the force-length and force-velocity relationships. In Hill-type models, the fibre behaviour is computed at each time such that the force produced by the muscle fibre is equal to the force produced by the SEE. The use of SS T-A definition leads to decrease the variation of fibre length and velocity. According to the force-velocity relationship, this decrease of shortening velocity due to less stiff definition of the SEE increases the corresponding muscle force for the same muscle activation (Fig. 8). The decrease in fibre length moves the muscle fibre closer to its optimal force length relationship and hence closer to maximal force. This different behaviour of muscle fibre obtained by using SS T-A definition explains the higher muscle force for the soleus.

The *in vivo* measurement of muscle forces is difficult due to the invasiveness of direct measurement methods (i.e surgical implants, etc…). In the present study, the validation of the estimation of muscle forces was not directly possible. Ultrasonography has been previously used to investigate the behaviour of the muscle fibre relative to the muscle-tendon unit. During walking task in humans, Ishikawa and al. [2] have shown the presence of a decoupling between muscle fascicle and muscle-tendon unit behaviours. During highly a dynamic task such as a drop jump, this decoupling is less important but remains present [2], [4]. The use of SS T-A definition leads to a lower muscle fibre velocity and a higher SEE velocity (i.e., a strong decoupling behaviour) compared to the use of generic definition. This higher decoupling behaviour between the muscle fibre and SEE is in agreement with previous experimental recordings. Sugisaki et al. [34] have combined motion capture and ultrasonography to investigate the behaviour of muscle fibre relative to the muscle-tendon unit for the GM muscle during dynamic contractions. Figure 9 illustrates the similarity of the behaviour of the SEE and the muscle fibre when using the SS T-A definition in a NMS model relative to the experimental measurements of Sugisaki et al. [34]. The comparison with experimental measurements highlights that the use of generic Zajac definition failed to represent a physiological behaviour of muscle fibre. The conformity of our results with previous experiments provides us with confidence in our modelling methods. We hypothesize that the higher predicted muscle forces from NMS models using a subject-specific SEE definition was a more physiologically accurate approach than the use of a generic definition.

### Perspectives

In this study, we were focused on the subject-specific tendon-aponeurosis force-strain relationship. Li et al. [35] have shown that the use of subject-specific optimal fibre length and pennation angle data improves the prediction of elbow movement for healthy individuals and stroke victims. The ultrasonography has also been used to estimate the subject-specific moment arm [36]. Further studies are necessary to investigate the influence of additional subject-specific muscle-tendon parameters into NMS models such as moment arm, and combined *in vivo* aponeurosis-tendon force-strain relationship, initial and optimal fibre length, and pennation angle.

## Conclusion

This study highlights the importance of using subject-specific measurements in terms of SEE definition in Hill-type muscle models during dynamic tasks. Subject-specific SEE induced a 17% increase in maximal predicted muscle force. Furthermore, it resulted in improved physiologically behaviour of the muscle fibre. Moreover, the present work combined direct experimental measurements to characterize the *in vivo* properties of the tendon-aponeurosis unit with the implementation of the results in a neuromusculoskeletal model to estimate muscle forces and fibre behaviours. Based on our results, the NMS model was able to numerically replicate experimental results showing a decoupling behaviour between the fibre and the muscle-tendon unit. The mechanical properties of tendon and aponeurosis may be altered by musculoskeletal disorders such as stroke [37], [38] or by ageing [39]. The present study demonstrates that it is crucial to take into account alterations of tendon-aponeurosis mechanical properties in order to accurately treat characterize muscle function.
